# Chronic Medical Conditions and Negative Affect; Racial Variation in Reciprocal Associations Over Time

**DOI:** 10.3389/fpsyt.2016.00140

**Published:** 2016-08-24

**Authors:** Shervin Assari, Maryam Moghani Lankarani

**Affiliations:** ^1^Department of Psychiatry, University of Michigan, Ann Arbor, MI, USA; ^2^Center for Research on Ethnicity, Culture and Health, School of Public Health, University of Michigan, Ann Arbor, MI, USA; ^3^Medicine and Health Promotion Institute, Tehran, Iran

**Keywords:** population groups, ethnic groups, African Americans, chronic medical conditions, depression

## Abstract

**Background:**

The Black–White health paradox can be defined as lower frequency of depression despite higher prevalence of economic and social adversities as well as chronic medical conditions (CMC) among American Blacks compared to American Whites. Based on this paradox, the CMC – depressive symptoms link is expected to be weaker among Blacks than Whites. We conducted a 10-year longitudinal study to compare Blacks and Whites for bidirectional associations between number of CMC and negative affect over time.

**Methods:**

We used data from the MIDUS (Midlife in the United States), a nationally representative longitudinal study of American adults. A total number of 7,108 individuals with an age range of 25–75 years (*N* = 7,108) were followed for 10 years from 1995 to 2004. Age, gender, and socioeconomic status (education and income) were measured at baseline. Negative affect and CMC were measured at baseline (1995) and end of follow up (2004). Race was the moderator. Linear regression was used to test the moderating effect of race on the reciprocal associations between CMC and negative affect, net of covariates.

**Results:**

In the pooled sample, while baseline CMC was predictive of an increase in negative affect over time, baseline negative affect was also predictive of an increase in CMC. We found interactions between race and baseline CMC on change in depressive symptoms, as well as race with negative affect on CMC change, suggesting that the associations between CMC and negative affect are stronger for Whites in comparison to Blacks.

**Conclusion:**

Blacks and Whites differ in reciprocal links between CMC and negative affect over time. This finding replicates recent studies on differential links between psychosocial factors and physical health based on race. Findings may help us better understand how Black-White health paradox develops across mid and later life.

## Introduction

Chronic medical conditions (CMC) are more prevalent among Blacks than Whites (1, 2). Emotional problems such as depression are, however, less common among Blacks (3–5). As a result, a larger discrepancy exists between CMC and depression among Blacks (6, 7). Disproportionately lower prevalence of depression despite higher levels of social and economic adversities as well as CMC among Blacks, compared to Whites, is also known as the Black–White health paradox (5, 6). Studies that have found weaker associations between CMC and depression among Blacks (8–11) have lent empirical support to the Black–White health paradox. Not all studies, however, have found such group differences (11–14).

As most prior studies in this area have used a cross-sectional design (9–14), there is still a need for additional longitudinal research (7). In 2015, Assari and colleagues applied multi-group structural equation modeling to data from the Americans’ Changing Lives (ACL) study and showed that among White but not Black respondents, higher CMC at baseline predicted an increase in depressive symptoms over a 25-year follow up period. Among Whites but not Blacks, depressive symptoms at baseline were predictive of incident CMC over time, as well (7). In another attempt, Assari and colleagues used multi-group cross-lagged modeling and documented race by gender group differences in the lagged effects of depressive symptoms and restless sleep on change in CMC over time. While White men, White women, and Black women were similar in the residual effect of restless sleep over depressive symptoms on CMC, such effect was missing among Black men (15).

These studies all suggest that contextual factors such as race and ethnicity should be conceptualized as moderators that may alter the reciprocal associations between CMC and depression (16–18). Also called as *the differential effect hypothesis*, this approach focuses on the contextual (moderating) effects of race and ethnicity, rather than their main (direct) effects. In this view, populations differ for associations that ultimately influence health and illness (16–18).

Despite our knowledge of the bidirectional nature of the relationship between depression and CMC, existing information is still limited on potential population differences in the reciprocal relationships between CMC and negative affect over time. Built on the existing literature (7, 11, 12, 19–22), we compared Blacks and Whites for the reciprocal associations between CMC and negative affect over a 10-year period.

## Materials and Methods

### Design and Setting

Data came from the MIDUS, 1995–2004. The MIDUS is a nationally representative longitudinal cohort study of 7,000 + adult Americans aged 25–74. The study is carried out by the MacArthur Midlife Research Network. The main purpose of the study was to investigate the role of psychosocial factors in understanding age variation in physical and mental health (23–26).

### Ethics

The study was approved by the Institutional Review Board at University of Wisconsin-Madison, UCLA, and Georgetown University. Informed written consent was obtained for all participants. The study was funded by the National Institute on Aging. Monetary incentives were offered at both wave 1 and 2 to compensate for potential respondent burden (US$20 for completion of MIDUS 1 surveys and up to US$60 for completion of MIDUS 2 surveys).

### Process

The survey was multimodal, composed of phone interviews and self-report questionnaires. First, the study employed an initial 30-min phone or face-to-face interview followed by a set (two) of self-administered questionnaires (SAQs). Questionnaires were mailed to individuals after completing the phone interview.

Data collection of the wave 1 was conducted in 1995 and 1996. Data collection of the wave 2 of the MIDUS was conducted in 2004 and 2005. The time-2 data collection used the original protocol (wave 1). All participants received letters as a reminder that an interviewer would contact them for a telephone survey in few weeks. Following completion of a phone interview that lasted 30-mins on average, participants received two SAQs *via* mail.

### Participants and Sampling

The study used random digit dialing (RDD) to enroll participants. RDD is a widely accepted method for recruiting people to telephone statistical surveys. RDD generates telephone numbers at random, thus MIDUS enrolled a random sample of adults. The study used telephone numbers within the continental United States as the sampling frame for the national RDD survey. The study also used an oversampling in five cities. This stage resulted in 4,244 individuals enrolled *via* RDD sampling. By randomly selecting 529 cases from the RDD sample who had at least one sibling, a sibling sample was generated. Siblings were limited to those within a family that had the same biological mother and father. Using this strategy, the study enrolled 950 siblings. The study also used a two-part sampling design to enroll the twin sample [957 twin pairs (*n* = 1,914)].

### Follow-up Data

From the total 7,108 participants who started the study (completing the phone survey at MIDUS 1), data were collected at wave 2 for 4,963 (70%) at MIDUS 2, which was 9–10 years later. Thus the MIDUS overall retention rate is 75% (adjusted for mortality). The retention rate was higher for siblings (81%) and twins (81%) compared to the RDD sample (69%). Major causes for non-participation at MIDUS 2 included refusal (12%), could not be contacted (10%), and too ill to be interviewed (8%) or deceased (verified by the National Death Index).

### Measures

We collected data on core demographic variables and socioeconomic factors at baseline. Depressive symptoms and CMC were measured at waves 1 and 2.

#### Socioeconomic Factors

Demographic variables were collected at baseline in 1995 and included age (continuous), gender (0 = *male*, 1 = *female*), and race (0 = *Whites*, 1 = *Blacks*). Socioeconomic variables included educational attainment (treated as a continuous measure; 1 = *less than high school*, 2 = *high school graduate or equivalent*, 3 = *some college*, 4 = *college graduate or more*) and personal income (continuous measure).

#### Chronic Medical Conditions

Data were collected at wave 1 and 2 on history of 20 CMC. Examples of conditions are cancer, heart disease, diabetes (high blood sugar), asthma, bronchitis or emphysema, ulcer, migraine, and thyroid disease (27, 28).

#### Negative Affect

State negative affect (SNA) was assessed using a 6-item questionnaire that asked participants how much of the time over the past 30 days did they feel: “so sad,” “nervous,” “restless or fidgety,” “hopeless,” “worthless,” and “everything was an effort.” Participants rated their response on a 5-point scale from 1 (none of the time) to 5 (all of the time). We calculated a mean score, which could range from 1 to 5, with higher score indicating more negative affect. In this measure, SNA is conceptualized as symptoms of depression and anxiety, two emotions commonly used to define negative affect (29–31). The scale was similar to K-6 developed by Kessler and colleagues, which has yielded a single factor structure representing current, general psycho-logical distress (32). Cronbach’s alpha ranged from 0.75 to 0.85 across the eight administrations of this scale (33). Internal consistency (reliability) was excellent (α = 0.86 for Whites and 0.87 for Blacks).

### Statistical Note

Data analysis was conducted in SPSS 20.0 for Windows (IBM Inc. Armonk, NY, USA). We ran multiple linear regressions in the pooled sample as well as based on race, with and without interaction. In the first step, CMC was the independent variable, and negative affect was the dependent variables. Then in the second step, negative affect was the independent variable, and CMC was the dependent variable. Race was the focal moderator. Age, gender, education, and income were covariates. Regression coefficients (b), SE with 95% Confidence Intervals (CI) were reported.

## Results

Table 1 presents the results of descriptive analysis in the pooled sample and based on race. Compared to Whites, Blacks were younger, were more frequently women, and had lower education and income. Blacks had higher CMC at wave 1 and 2 compared to Whites. Blacks also had higher negative affect at wave 2.

**Table 1:** **Descriptive Statistics in the pooled sample and based on race**.

	All		Whites		Blacks	
	*n*	%	*n*	%	*n*	%
Gender*						
Men	3395	47.8	2683	47.9	121	37.7
Women	3632	51.1	2917	52.1	200	62.3

	**Mean**	**SD**	**Mean**	**SD**	**Mean**	**SD**

Age*	46.38	13.00	47.30	12.92	44.42	12.54
Education*	6.77	2.49	6.90	2.47	6.22	2.47
Income*	26773.24	26891.19	27326.10	27509.71	20762.54	19730.37
Chronic medical conditions (wave 1)*	2.41	2.51	2.39	2.46	2.53	2.96
Chronic medical conditions (wave 1)*	2.46	2.59	2.39	2.41	3.16	4.56
Negative affect (wave 1)	1.54	0.62	1.53	0.61	1.53	0.65
Negative affect (wave 2)*	1.51	0.58	1.50	0.56	1.67	0.83

*p < 0.05.

Table 2 summarizes two regression models in the pooled sample with CMC as the predictor and depressive symptoms as the outcome. According to *Model 1*, which did not include the interaction term, baseline CMC predicted change in negative affect, net of socioeconomic factors. *Model 2* also included an interaction between race and CMC showed a significant and negative interaction between Black race and baseline CMC on change in negative affect, suggesting that the association between baseline CMC and subsequent change in negative affect is stronger for Whites in comparison to Blacks.

**Table 2:** **Predictive role of baseline chronic medical conditions on wave 2 negative affect in the pooled sample**.

	*B* (SE)	95% CI	*B* (SE)	95% CI
	
	Model 1 Pooled Sample, No Interaction		Model 2 Pooled Sample, With Interaction	
Race (Blacks)	0.96 (1.35)	−1.70–3.61	3.61 (1.88)^#^	−0.08–7.30
Age	−0.05 (0.02)**	−0.09–0.01	−0.05 (0.02)*	−0.09–0.01
Gender	−0.81 (0.47)^#^	−1.72–0.11	−0.79 (0.47)^#^	−1.70–0.12
Education	−0.16 (0.09)^#^	−0.35–0.02	−0.15 (0.09)	−0.34–0.03
Income	−0.01 (0.00)*	0.00–0.00	−0.01 (0.00)*	0.00–0.00
Negative affect (wave 1)	6.15 (0.44)***	5.29–7.00	6.18 (0.44)***	5.33–7.04
Chronic medical conditions (wave 1)	0.27 (0.11)*	0.06–0.49	0.31 (0.11)**	0.09–0.53
Chronic medical conditions (wave 1) × Blacks	–	–	−1.04 (0.51)*	−2.05–0.03

*^#^p < 0.1*.

*p < 0.05.

**p < 0.01.

***p < 0.001.

Table 3 provides a summary of two regression models in the pooled sample with negative affect at wave 1 as the predictor and CMC at wave 2 as the outcome. According to our *Model 1* which did not include the interaction term, baseline negative affect was predictive of change in CMC, net of socioeconomic factors. Based on *Model 2*, there was a significant interaction between race and baseline negative affect on CMC at wave 2, suggesting that race alters the effect of baseline negative affect and change in CMC.

**Table 3:** **Predictive role of baseline negative affect on wave 2 chronic medical conditions in the pooled sample**.

	*B* (SE)	95% CI	*B* (SE)	95% CI
	
	Model 1 Pooled Sample, No Interaction		Model 2 Pooled Sample, With Interaction	
Race (Blacks)	0.51 (0.18)**	0.16–0.87	−1.24 (0.86)	−2.93–0.44
Age	0.03 (0.00)***	0.02–0.03	0.03 (0.00)***	0.02–0.03
Gender (Women)	0.24 (0.07)***	0.09–0.38	0.24 (0.07)***	0.10–0.39
Education	−0.06 (0.01)***	−0.09–0.03	−0.06 (0.01)***	−0.09–0.03
Income	−0.01 (0.00)	0.00–0.00	−0.01 (0.00)	0.00–0.00
Negative Affect (Wave 1)	0.32 (0.06)***	0.20–0.45	0.34 (0.06)***	0.21–0.46
Chronic Medical Conditions (Wave 1)	0.49 (0.02)***	0.46–0.52	0.49 (0.02)***	0.46–0.52
Negative Affect (Wave 1) × Blacks	–	–	0.49 (0.24)**	0.03–0.96

*^#^p < 0.1*.

**p < 0.01.

***p < 0.001.

Table 4 presents the results of regression models with CMC at wave 1 as the independent variable and negative affect at wave 2 as the outcome across race groups. Only among Whites (*Model 1*) but not Blacks (*Model 2*), baseline CMC predicted a change in negative affect over time, net of socioeconomic factors.

**Table 4:** **Predictive role of baseline chronic medical conditions (1995) on subsequent negative affect (2004) among Whites and Blacks**.

	*B* (SE)	95% CI	*B* (SE)	95% CI
	
	Model 1 Whites		Model 2 Blacks	
Age	−0.05 (0.02)**	−0.09–0.01	0.01 (0.19)	−0.38–0.40
Gender (women)	−0.86 (0.47)^#^	−1.78–0.05	2.47 (4.58)	−7.15–12.09
Education	−0.14 (0.09)	−0.33–0.04	−0.42 (1.02)	−2.56–1.71
Income	−0.01 (0.00)*	0.00–0.00	−0.01 (0.00)	0.00–0.00
Negative affect (Wave 1)	6.00 (0.44)***	5.12–6.87	9.01 (2.67)**	3.41–14.62
Chronic medical conditions (Wave 1)	0.34 (0.11)**	0.12–0.55	−1.34 (0.89)	−3.22–0.53

*^#^p < 0.1*.

*p < 0.05.

**p < 0.01.

*** p < 0.001.

Table 5 summarizes the results of race-specific regression models with negative affect at wave 1 as the predictor and CMC at wave 2 as the outcome. Among Whites (*Model 1*), baseline negative affect predicted CMC change over time, net of demographic and socioeconomic factors. The same association could not be found for Blacks (*Model* 2).

**Table 5:** **Predictive role of baseline negative affect (1995) on subsequent chronic medical conditions (2004) among Whites and Blacks**.

	*B* (SE)	95% CI	*B* (SE)	95% CI
	
	Model 1 Whites		Model 2 Blacks	
Age	0.03 (0.00)***	0.02–0.03	0.03 (0.03)	−0.03–0.09
Gender (women)	0.20 (0.07)**	0.06–0.34	1.31 (0.79)^#^	−0.25–2.88
Education	−0.04 (0.01)**	−0.07–0.01	−0.44 (0.14)**	−0.71–0.16
Income	−0.01 (0.00)^#^	0.00–0.00	0.25 (0.00)**	0.00–0.00
Chronic medical conditions (Wave 1)	0.51 (0.02)***	0.48–0.54	0.25 (0.14)^#^	−0.02–0.53
Negative affect (Wave 1)	0.33 (0.06)***	0.21–0.46	0.08 (0.50)	−0.91–1.06

*^#^p < 0.1*.

**p < 0.01.

***p < 0.001.

## Discussion

According to our findings, Whites and Blacks differ in reciprocal and longitudinal associations between CMC and negative affect over a 10-year period. Thus, race alters the inter-relation between baseline and change of CMC and negative affect in the United States.

In our study, baseline CMC predicted change in negative affect among Whites but not Blacks. Similarly, baseline negative affect predicted change in CMC among Whites but not Blacks. These findings replicate a previous report by Assari et al., using 25-year follow-up data (7). This replication is important as Assari et al. mentioned: “… *the duration of follow up was 25 years, which may have caused selective deaths in the population, particularly among Blacks and those with high baseline chronic medical conditions. Future reports may consider replication of the current findings using shorter follow-up periods.”* (7). These studies provide longitudinal support for the Black–White health paradox (6, 7, 22, 34, 35), defined as lower prevalence of depression or depressive symptoms (4) despite higher prevalence of CMC and other social and economic adversities (1, 2).

Capistrant and colleagues have shown that the link between baseline depressive symptoms and subsequent mortality due to cardiovascular causes was significant for Whites but not Blacks, after adjusting for potential confounders (36). There are other studies that have documented racial differences in depression associated with specific CMC types (20, 21, 37). For instance, Lewis and colleagues found evidence suggesting that depressive symptoms may have different effect on cardiovascular risk for Blacks and Whites (8, 38).

There are multiple explanations for Black–White differences in the link between depression and CMC. Jackson and colleagues have attributed these findings to racial differences in engagement in a number of unhealthy behaviors such as smoking or overeating that help Blacks cope with psychological distress but impose physiological damages at the same time (37, 39). Such ineffective coping behaviors may increase the physical toll associated with stress and distress among Blacks (19, 37, 39–41).

Literature has also provided results that are either inconsistent (11, 12, 37) or unexpected (10, 11, 42). As compared to Whites, Blacks have more CMC but less depression, we intuitively expect weaker depressive symptoms – CMC link for Blacks. That is, any incremental increase in CMC is expected to have a smaller effect on depressive symptoms among Blacks compared to Whites. Hankerson and colleagues, however, documented a stronger association between major depressive disorder (MDD) and hypertension, obesity, and liver disease among Blacks compared to Whites (42), and Assari found cross-sectional positive association between MDD and cardiovascular diseases among Blacks that could not be replicated for Whites (10). Further, using the National Survey of American Life (NSAL) data, Watkins, Assari, and Johnson-Lawrence showed that lifetime MDD was associated with at least one CMC among Blacks, but not Whites (12). A few existing studies have failed to show any moderating effect of race on the association between CMC and depression (11, 14). As several of the above findings contradict the expected stronger association between depression and CMC among Whites than Blacks, we still need more research that explores racial variations in the cross-sectional and longitudinal links between CMC and depression.

Previous research has shown that the link between depression and CMC is bidirectional (43–49). While baseline CMC predicts subsequent depression (43–47), baseline depression also increases risk of CMC in the future (48, 49). However, this literature has mainly enrolled White middle class individuals (43, 47–49) and more is yet to be known on population variations in these associations (50).

Depression is known to be more chronic and disabling for Blacks in comparison to Whites (10, 12). Among those with lifetime MDD, frequency of 12-month MDD is higher for Blacks (56%) than Whites (39%) (4). Higher chronicity of depression for Blacks (4, 10, 12) may be in part due to low access and trust to the health care system as well as high stigma for Blacks than Whites (4, 10, 12). Negative beliefs regarding pharmaceutical treatment (4), preference of non-pharmacologic approaches (e.g., counseling and prayer) (51), and the belief that antidepressants are addictive (51) may also operate as barriers against depression treatment among Blacks. Higher comorbidity of CMC among Blacks with depression also adds to the complexity of diagnosis of depression among Blacks (1, 2, 52, 53). Differential presentation of depression (more somatic among Blacks) may also be another barrier for diagnosis (54). Given the worse trajectory of depression among Blacks, one would expect stronger effects of depression and negative emotions on CMC for Blacks (7).

Improving screening, diagnoses, and treatment of emotional problems such as depression among Blacks require major investment (4, 55, 56). Still, the primary care setting should be regarded as a unique opportunity for depression screening among Blacks who receive care for their medical conditions (57–59). However, quality improvement programs should also enhance quality of psychiatric services in primary care settings, particularly for Blacks (12, 60).

This study contributes to a growing literature that has documented Black–White differences in how psychological factors contribute to a wide range of physical health outcomes including but not limited to CMC and mortality (7, 61, 62). Lucas and colleagues have shown that psychological as well as biological processes that contribute to cardiovascular health disparities are not simply the consequence of individual level exposures but the interplay between individual-level and contextual factors (e.g., views about justice) (63). Mezuk et al. have suggested that behaviors may explain why the links between emotional and physical health problems are weaker among Blacks (41). Keyes et al. have attributed such differences to flourishing of Blacks in the face of social inequality and discrimination (5, 6). Kitayama et al. have attributed these findings to cultural differences in tolerance and affordance of emotional dysregulation (64–66). Other researchers have attributed these group differences to stereotypes and schemas such as strong Black Woman ideology (67–69). Such differential effects are not specific to depression and negative affect (16) and have been seen for self-rated health as well (17). These findings are also consistent with findings by Stewart and colleagues that suggest depressive symptoms predict inflammatory markers among Whites but not Blacks (70–72). Boyle, Williams, and others have also shown Black–White differences in the link between psychological and biological markers of serotonin with insulin sensitivity (73–76).

The results reported here should be interpreted with the following limitations in mind. First, this study measured number of CMC, regardless of their type. Second, CMC measurement was based on self-reported data. As race may alter associations between depression and particular types of CMC (8, 9, 14, 20, 21, 37, 38, 43), there is a need to determine CMC types that may differently correlate with depression and negative emotions based on race. Third, this study measured negative affect, not clinical depression. Some studies have suggested that clinical diagnosis of depression is differently associated with CMC than depressive symptoms (8, 9, 14, 20, 21, 37, 38, 43). Negative emotions may also differently predict MDD across racial groups (70). The study did not control for access to health care, insurance, and function, as well as behaviors that may influence CMC, depression, or both. Forth, Whites were over-represented in the sample. Despite these limitations, this study still makes a unique contribution to the literature as only a handful of studies have compared Blacks and Whites for reciprocal associations between affect and CMC over time (7).

Our findings advocate for designing and implementing tailored rather than universal programs that screen for combined physical and emotional health problems at the community as well as in primary health-care settings. As racial groups differ in how physical and mental health problems co-exist and develop, race should be in the center of comprehensive protocols that focus simultaneously on multiple health aspects of diverse populations. Race groups with similar level of CMC may have different emotional needs. Integrative care may particularly benefit from adjustment to culture, race, and ethnicity (77). Findings may also have implications for the health-care reform, also known as Obama care (78).

In summary, we found racial differences in reciprocal associations between CMC and negative affect over a 10-year period. Comorbidity between physical and emotional health problems is not independent of context, and the inter-relations between the trajectories of these health problems may be context specific.

## Author Contributions

SA was responsible for the design and analysis of the data and revisions. ML drafted the manuscript. Both authors approved the final draft.

## Conflict of Interest Statement

The authors declare that the research was conducted in the absence of any commercial or financial relationships that could be construed as a potential conflict of interest.
